# Outcomes of palliative radiation therapy for cutaneous squamous cell carcinoma: A retrospective cohort study

**DOI:** 10.1016/j.jdin.2023.09.008

**Published:** 2023-10-06

**Authors:** Emily Y. Kim, Emily E. Granger, Jonathan D. Schoenfeld, Ann W. Silk, Danielle Margalit, Roy Tishler, Emily S. Ruiz

**Affiliations:** a Department of Dermatology, Brigham and Women’s Hospital, Boston, Massachusetts; b Department of Radiation Oncology, Dana-Farber Cancer Institute and Brigham and Women's Hospital, Boston, Massachusetts; c Harvard Medical School, Boston, Massachusetts; d Department of Medical Oncology, Dana-Farber Cancer Institute, Boston, Massachusetts

**Keywords:** oncology, palliative, radiation, radiotherapy, squamous cell carcinoma

*To the Editor:* Palliative radiation therapy (PRT) can be utilized for advanced, unresectable cutaneous squamous cell carcinoma (cSCC) for symptomatic and/or locoregional control. Data on outcomes of PRT for skin cancer are limited.^1^^,^^2^ There are 2 studies on PRT in cSCC; however, 1 includes patients treated with both curative or palliative intent and does not present details on disease characteristics or outcomes (*n* = 14).^3^ A second study of PRT for 12 patients focuses on factors associated with improved overall survival (OS), but does not report disease-specific survival (DSS), symptomatic control, or in-field response.^4^ Given the lack of long-term data in cSCC, this retrospective cohort study evaluates the utility of PRT for advanced cSCC.

Institutional data retrieval system was queried for patients treated with palliative-intent radiotherapy for symptomatic locoregionally advanced, unresectable cSCC between January 1, 2014 and December 31, 2020 (*n* = 14). Tumors impinging on important anatomical structures and/or tumors for which surgery would lead to unacceptable functional deficits were considered unresectable. PRT was defined as radiation intended for symptom relief and improvement of quality of life. This study was approved by our institutional review board.

Primary outcomes were rate of symptomatic control, 1-, 2-, 3-year OS, DSS, and in-field progression-free survival (PFS). OS and DSS were calculated from date of first fraction to date of death from any or cSCC-related causes, respectively. PFS was calculated from date of first fraction to date of in-treatment field progression or recurrence determined by a board-certified dermatologist or radiation oncologist.

Table I includes patient, tumor, and treatment characteristics. PRT doses ranged from 30 to 70 Gy over 5-35 fractions. All patients had American Joint Committee on Cancer Eighth Edition (AJCC-8) stage III/IV disease.^5^ Median DSS was 8.1 months (95% CI 3.0-N/A). 1-year DSS rate was 49.1% (95% CI 19.0%-73.7%); 2- and 3-year DSS rates were 36.8% (95% CI 10.2%-64.6%) (Fig 1). Median OS was 5.0 months (95% CI 3.0-12.6). One-year OS rate was 28.6% (95% CI 8.8%-52.4%); 2- and 3-year OS rates were 21.4% (95% CI 5.2% to 44.8%) (Supplementary Fig 1, available via Mendeley at https://doi.org/10.17632/z874fnhxhp.1). Median time to in-field recurrence or progression was 4.5 months (95% CI 1.6-N/A), with only 2 patients (14.0%) experiencing PFS beyond 1 year (Supplementary Fig 2, available via Mendeley at https://doi.org/10.17632/yc43f292wx.1). Eight patients (57.1%) had in-field symptomatic control. One (7.1%) patient developed in-field recurrence after complete response, 1 (7.1%) developed progression elsewhere, and 1 (7.1%) developed both in-field progression and distant metastases. Five (35.7%) patients had in-field disease progression. Six patients (42.9%) had a favorable response or stable disease without progression after treatment. Mean EQD2 was similar between patients who recurred/progressed and those who did not (45.5 vs 50.0, *P* = .6).

**Table I:** Patient, tumor, and treatment characteristics

Median age – year (range)	83 (59-99)
Male sex – no. (%)	10/14 (71)
ECOG – no. (%)	
0	1/14 (7)
1	6/14 (43)
2	3/14 (21)
3	4/14 (29)
Immunosuppression, solid organ transplant – no. (%)	2/14 (14)
Primary tumor location – no. (%)	
Head/neck	8/14 (57)
Trunk/extremities	2/14 (14)
Nodal or parotid disease only	4/14 (29)
Staging group (AJCC 8)∗ – no. (%)	
III	4/14 (29)
IV	10/14 (71)
Disease level at presentation† – no. (%)	
Unresectable local	9/14 (64)
Unresectable nodal	11/14 (79)
In-transit metastases	4/14 (29)
Distant metastases	2/14 (14)
Recurrent disease at presentation – no. (%)	9/14 (64)
Radiation target – no. (%)	
Symptomatic primary tumor	3/14 (21)
Symptomatic nodal tumor	5/14 (36)
Symptomatic primary tumor and draining lymph nodes	6/14 (43)
Median RT dose – Gy (range)	39.5 (30-70)
Median number of fractions – no. (%)	13 (5-35)
Median biologically effective dose – Gy (range)	50.7 (19-89.7)
Median equivalent dose (EQD2) – Gy (range)	42.2 (15.8-74.7)
Radiation technique	
IMRT/VMAT	5/14 (36)
Electron beam	3/14 (21)
Other	6/14 (43)
Concurrent treatment – no. (%)	
Immunotherapy	1/14 (7)
Cisplatin	1/14 (7)
Cetuximab	4/14 (29)
RT only	8/14 (57)
Radiation outcome‡ – no. (%)	
Symptom control	8/14 (57)
Complete response of tumor	3/14 (21)
Persistent/stable disease	4/14 (29)
Disease progression or recurrence in treatment field	6/14 (43)
Disease progression outside treatment field	1/14 (7)

∗ Primary tumor stage (AJCC 8): 4/14 (29%) TX, 1/14 (7%) T1, 2/14 (14%) T2, 4/14 (29%) T3, 3/14 (21%) T4. Primary tumor stage (BWH): 1/14 (7%) T1, 6/14 (43%) T2b, 3/14 (21%) T3, 4/14 (29%) not evaluable. Nodal stage (AJCC 8): 3/14 (21%) N0, 1/14 (7%) N1, 3/14 (21%) N2b, 7/14 (50%) N3. Poorly differentiated tumors 8/14 (57.1%). Perineural invasion 4/14 (28.6%). Lymphovascular invasion 3/14 (21.4%).

† Numbers add up to more than 100 because patients can contribute to more than one category.

‡ Numbers add up to more than 100 because patients with symptom control contributed to more than one category.

**Fig 1:**
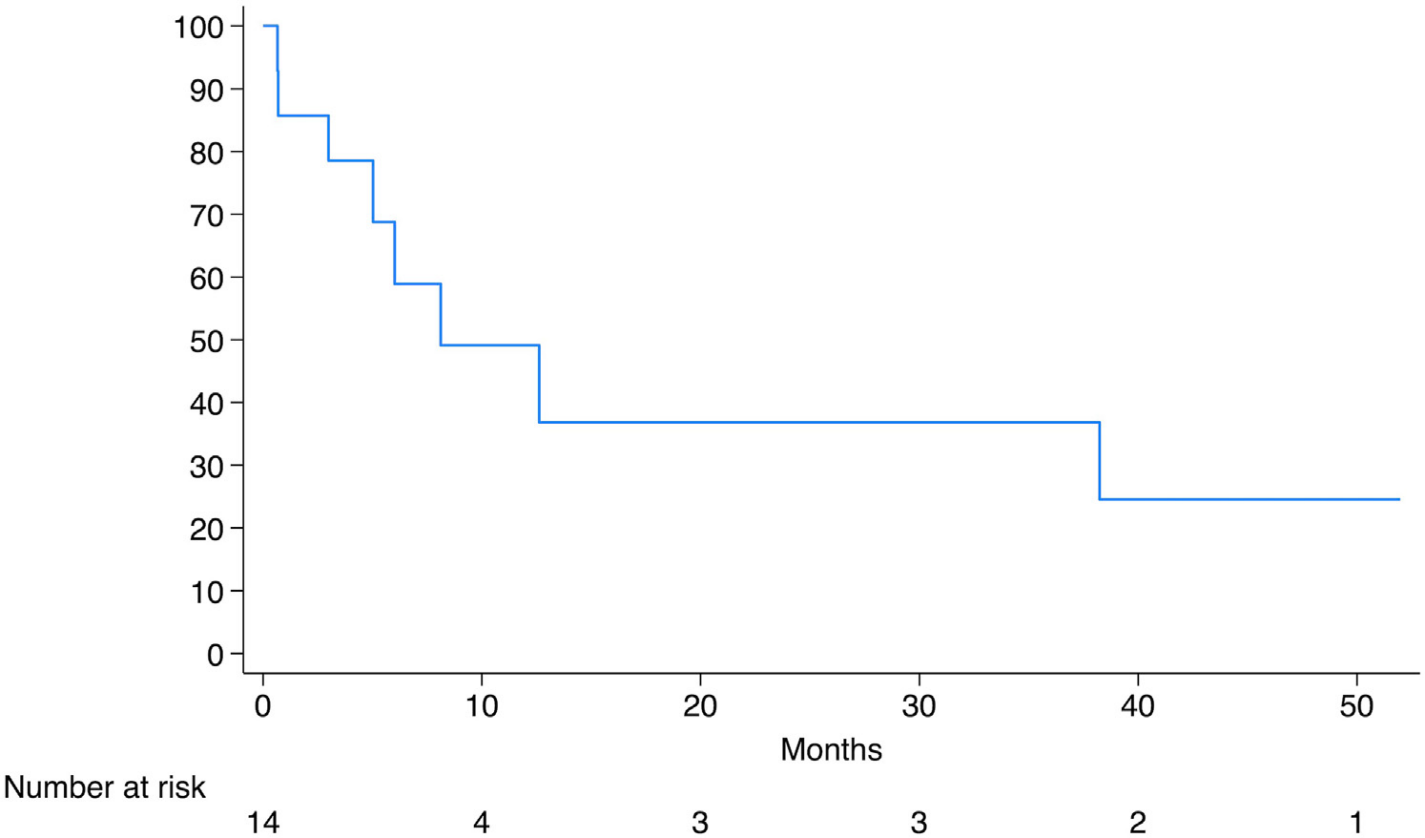
Kaplan-Meier curve depicting disease-specific survival for 14 patients treated with palliative radiation therapy for symptomatic locoregionally advanced, unresectable cutaneous squamous cell carcinoma.

Dermatologists should consider referral for PRT for tumors causing significant pain or functional impairment. In this study on long-term outcomes of PRT for symptomatic locoregionally advanced, unresectable cSCC, half of patients had in-field symptomatic control with complete or partial response, or disease stability. Future studies should define the optimal PRT regimen and whether concurrent systemic therapy provides greater benefit. A better understanding of the role of PRT will improve the quality of life of patients who have limited management options for advanced cSCC.

## Conflicts of interest

Dr Schoenfeld reported receiving research support paid to the institution from Merck, BMS, Regeneron, Debiopharm, EMD Serono; Consulting/Scientific Advisory Board/Travel fees from Castle Biosciences, Genentech, Immunitas, Debiopharm, ACI Clinical, Astellas, Stimit, Merck KGA, SIRPant, EMD Serono; stock options from Immunitas, and equity from Doximity. No other disclosures were reported.

## References

[1] Barnes E.A., Breen D., Culleton S. (2010). Palliative radiotherapy for non-melanoma skin cancer. Clin Oncol (R Coll Radiol).

[2] Kim S.K., Barker C.A. (2018). Outcomes of radiation therapy for advanced T3/T4 non-melanoma cutaneous squamous cell and basal cell carcinoma. Br J Dermatol.

[3] Haehl E., Rühle A., Klink R. (2021). The value of primary and adjuvant radiotherapy for cutaneous squamous cell carcinomas of the head-and-neck region in the elderly. Radiat Oncol Lond Engl.

[4] Staackmann C., Schild S.E., Rades D. (2021). Palliative radiotherapy for cutaneous squamous cell carcinoma of the head-and-neck region. In Vivo.

[5] Amin M.B., Greene F.L., Edge S.B. (2017). The Eighth Edition AJCC Cancer Staging Manual: continuing to build a bridge from a population-based to a more “personalized” approach to cancer staging. CA Cancer J Clin.

